# Note from the editors: World Tuberculosis Day 2018 and Special issue–Screening and prevention of infectious diseases in newly arrived migrants in Europe

**DOI:** 10.2807/1560-7917.ES.2018.23.12.180322-1

**Published:** 2018-03-22

**Authors:** Eurosurveillance editorial team

**Affiliations:** 1 European Centre for Disease Prevention and Control (ECDC), Stockholm, Sweden

**Keywords:** tuberculosis, screening, migrants, Europe

World Tuberculosis Day (WTBD) on 24 March commemorates the day in 1882 when Robert Koch presented his discovery of the bacterium that causes tuberculosis (TB). It is a good opportunity to raise awareness about the still-existing burden of TB worldwide and in Europe.

According to the latest figures published on 19 March 2018 in the TB surveillance report by the European Centre for Disease Prevention and Control (ECDC)/World Health Organization (WHO) Regional Office for Europe, the European Union and European Economic Area (EU/EEA) countries reported 58,994 TB cases in 2016 and that the notification rate has declined by 4.5% in the last 5 years [1]. While this is good news in principle, this annual rate of decline is unfortunately still insufficient to achieve the United Nations’ Sustainable Development Goal target of ending TB by 2030 [2]. Furthermore, resistance against the drugs to cure TB poses a problem in Europe. The rate of notified multidrug-resistant (MDR) TB cases in the EU/EEA has remained unchanged at 0.3 per 100,000 population since 2012, but the proportion of extensively drug-resistant (XDR) TB cases among MDR TB cases increased from 13.9% to 20.6%. For both MDR TB and XDR TB, treatment success rates are below the 75% target set for the WHO European Region by 2020 [3]. Initiatives have been started to further improve the TB situation in the EU/EEA and to address the threat of TB and MDR TB. For example, a pilot project by the ECDC on the use of whole genome sequencing (WGS) that will establish common standards and build capacity for WGS to improve the detection and investigation of TB outbreaks [4]. At the global level, the United Nations General Assembly will for the first time hold a high-level meeting on TB in September this year to discuss actions to accelerate the pace of TB elimination.

To mark WTBD 2018, *Eurosurveillance* published the first articles of the ‘Special issue–Screening and prevention of infectious diseases in newly arrived migrants in Europe’ on 15 March 2018 and this week’s issue features a further article from the series. In total, eight contributions were selected for the special issue from the 24 manuscripts submitted in response to a call for papers in March 2017 [5]. They will be published successively in the coming weeks and the whole series will be made available for download as a single PDF thereafter. Five of the articles cover matters pertaining to TB. The ones published so far are on TB treatment outcomes among screened newly-arrived asylum seekers in Germany [6], the acceptance of integrating hepatitis B, hepatitis C and HIV screening into TB entry screening for migrants in the Netherlands [7], and the outcome of TB screening asylum seeking children and adolescents from countries with high and low TB prevalence in reception centres in two German cities [8]. Two systematic reviews on the effectiveness and cost-effectiveness of screening for active and latent TB among migrants in the EU/EEA will follow [9,10] – stay tuned.
